# A Comparison of the Ultrasound-Guided Dorsal Penile Block and Ultrasound-Guided Caudal Epidural Block for Postoperative Analgesia in Children Undergoing Hypospadias Surgery: A Randomized Controlled Study

**DOI:** 10.7759/cureus.82351

**Published:** 2025-04-16

**Authors:** Snigdha Kumari, Mayank Kumar, Mamta Sinha, Subrata K Singha, Harishchandra Gupta, Rashmi Dubey

**Affiliations:** 1 Anesthesiology, Shri Balaji Institute of Medical Science, Raipur, IND; 2 Anaesthesiology, All India Institute of Medical Sciences, Raipur, Raipur, IND

**Keywords:** caudal epidural block, dorsal penile nerve block, hypospadias, postoperative pain, ultrasound

## Abstract

Introduction: Hypospadias repair is a common pediatric surgical procedure. While caudal epidural block (CB) is the standard analgesic technique, dorsal penile nerve block (DB) serves as a viable alternative. This study evaluated and compared the postoperative analgesic effectiveness of CB and DB in children undergoing hypospadias repair.

Methods: A total of 60 children aged one to six years undergoing hypospadias surgery were randomly assigned to either Group CB (ultrasound-guided caudal epidural block) or Group DB (ultrasound-guided dorsal penile block). The primary objective was to assess the time to first rescue analgesia, while the secondary objectives included evaluating postoperative pain using the Face, Legs, Activity, Cry and Consolability (FLACC) scale, postoperative analgesic requirements, and potential adverse effects such as hypotension, urinary retention, and respiratory depression.

Results: The time to first rescue analgesia was significantly longer in Group DB compared to Group CB (20.90 ± 6.49 vs. 11.48 ± 6.13 hours, p<0.001). Rescue analgesia requirement was significantly higher in Group CB (96.7%) than in Group DB (23.3%) within 24 hours (p<0.001). The average FLACC score was significantly higher in Group CB compared to Group DB (0.72 ± 0.29 vs. 0.25 ± 0.22, p<0.001). No complications were observed in either group.

Conclusion: Ultrasound-guided dorsal penile block proved more effective than caudal epidural block for postoperative pain management in hypospadias surgery, leading to longer time to first analgesic administration, lower pain scores, and reduced rescue analgesic requirements. Further studies are required to validate these findings.

## Introduction

Hypospadias, a common congenital anomaly, is marked by the urethral meatus opening on the ventral side of the penis at various levels, affecting approximately 1 in 125 live male births [1]. Various regional anesthetic techniques are commonly used to manage postoperative pain in hypospadias repair, including the caudal epidural block (CB), pudendal nerve block, sacral erector spinae block, and dorsal penile nerve block (DB) [2-4].

The caudal epidural block, a widely used neuraxial technique, is effective in providing postoperative analgesia in pediatric patients undergoing surgeries of the urogenital system [5]. Although the serious side effects of the caudal epidural block, such as urinary retention, motor block, intravascular or subarachnoid injection and complete spinal anesthesia, are rare, the role of CB in the formation of urocutaneous fistulas, one of the most concerning complications of hypospadias surgery, remains a topic of debate [6,7].

The dorsal penile nerve block is a peripheral nerve block technique that provides effective analgesia during and after penile surgeries [8]. Sandeman et al. demonstrated the efficacy of the landmark-based dorsal penile block in providing optimal postoperative pain relief in penile procedures [9]. Landmark-guided DB carries a risk of complications such as urethral injury, hemorrhage, hematoma formation, and ischemic glans [10]. Ultrasound-guided dorsal penile nerve block (US-guided DB) offers simplicity, safety, precise targeting, and improved tissue visibility, reducing associated challenges [2,11].

There is very limited literature comparing US-guided DB to US-guided CB for postoperative analgesia following hypospadias surgery. The primary objective of this study was to compare the analgesic efficacy of US-guided DB and US-guided CB techniques by evaluating the time until the first rescue analgesic was required. The secondary objectives included evaluating postoperative pain using the Face, Legs, Activity, Cry and Consolability (FLACC) scale, postoperative analgesic requirements, and potential adverse effects such as hypotension, urinary retention, and respiratory depression.

## Materials and methods

This randomized, double-blinded, parallel-group study was conducted in the Department of Anaesthesiology at All India Institute of Medical Sciences (AIIMS), Raipur, Chhattisgarh, India. The study received approval from the Institute Ethics Committee of AIIMS, Raipur (4190/IEC-AIIMSRPR/2023, dated December 19, 2023) and was registered with the Clinical Trials Registry of India (CTRI/2024/03/064112). Informed consent was obtained from the parents or guardians of all participants. Sixty male children aged one to six years with an American Society of Anesthesiologists (ASA) physical status of I or II who underwent hypospadias surgery under general anesthesia were included in the study. Exclusion criteria comprised parental refusal to consent, contraindications to regional anesthesia, history of developmental delay or intellectual disability that could hinder pain intensity assessment, and presence of any neurological abnormalities.

Sample size estimation

Based on the study conducted by Ahmed et al., the time to first rescue analgesia was 10.56 ± 4.67 hours in the caudal block group for hypospadias surgery [3]. Assuming a 25% longer time to first rescue analgesia with the penile nerve block, a study power of 80% and a 5% significance level, a minimum of 25 participants per group was required. To account for potential dropouts, we increased the sample size to 30 per group.

The study utilized a block randomization method with a computer-generated random number table. Participants were allocated into two groups, each comprising 30 patients. Allocation was concealed using sequentially numbered, opaque, sealed envelopes. On the day of surgery, the envelope for each recruited patient was opened by the anesthesiologist in the operating room immediately before administering the assigned block. To maintain blinding, a separate anesthesiologist, unaware of the block performed, assessed postoperative FLACC scores and analgesic requirements. Group CB received an ultrasound-guided caudal block with 0.5 ml/kg of 0.25% bupivacaine and Group DB received an ultrasound-guided dorsal penile block with 0.2 ml/kg of 0.25% bupivacaine.

Procedure

Detailed history taking and clinical examination were carried out and informed written consent was obtained from the patients’ parents. The entire anesthetic procedure, including the block techniques, was explained to the parents with relevant investigations carried out as per institutional protocol. Patients were kept nil per oral as per the ASA standard. In the operating room, standard ASA monitoring (electrocardiogram, non-invasive blood pressure, and pulse oximetry) was applied, and baseline parameters were documented.

Anesthesia was induced in a standardized manner using 6%-8% sevoflurane in 100% oxygen, followed by the placement of an appropriately sized intravenous cannula. Intravenous fentanyl (2 µg/kg) was given, followed by confirmation of adequate ventilation. Atracurium (0.5 mg/kg IV) was then given to facilitate the placement of a supraglottic airway device. Anesthesia was maintained with 1% sevoflurane and a mixture of 50% nitrous oxide in oxygen.

After induction, either an ultrasound-guided caudal epidural block or an ultrasound-guided dorsal penile nerve block was performed.

Caudal Epidural Block

**Figure 1:**
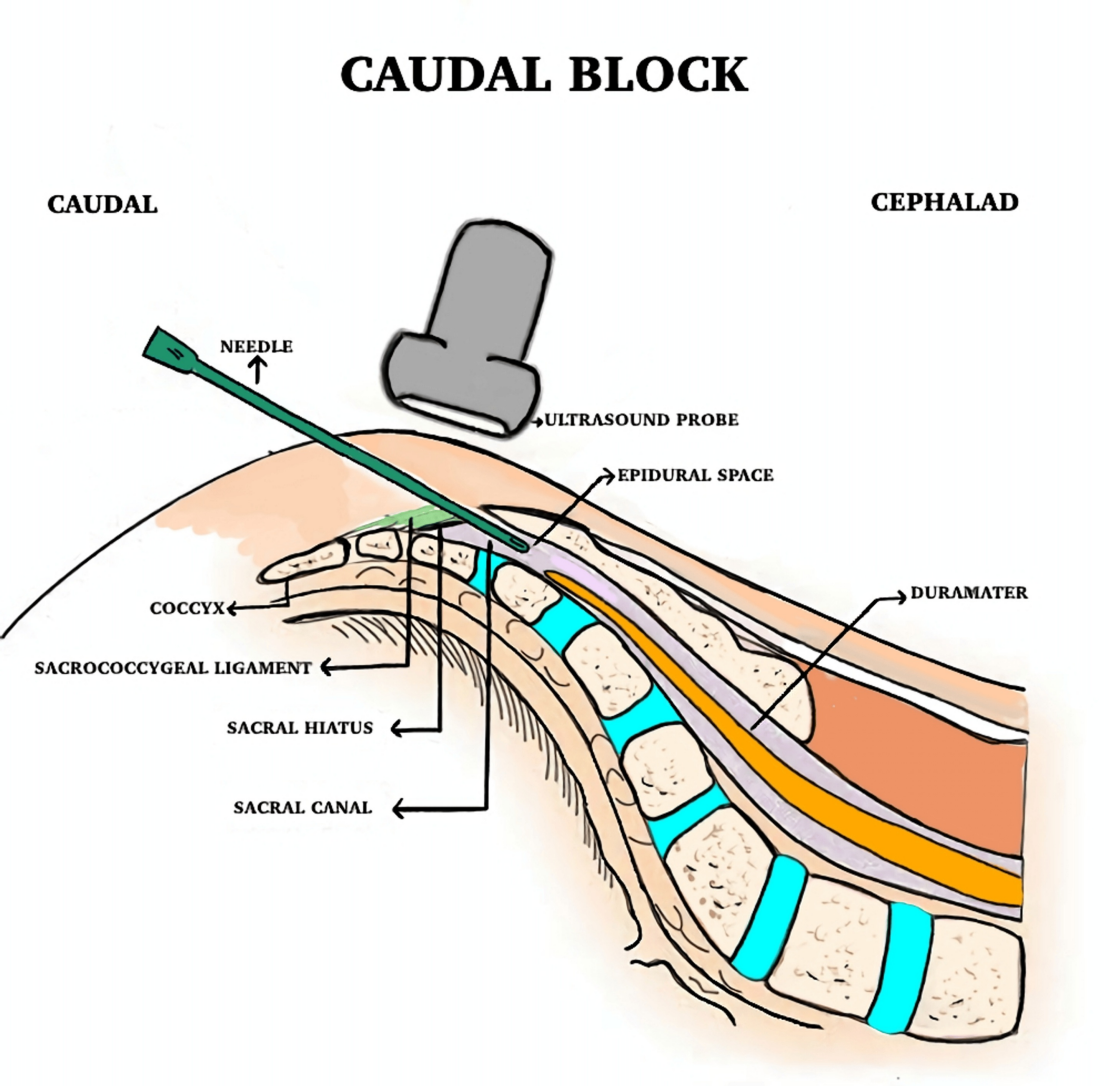
A schematic diagram of the caudal epidural block

In the CB group, patients were positioned laterally for the procedure. The ultrasound transducer was first positioned transversely at the midline to visualize the sacral cornua, sacrococcygeal ligament, sacral bone, and sacral hiatus in a transverse view. It was then rotated 90° to obtain a longitudinal view of the sacrococcygeal ligament and sacral hiatus. A 22G, 50 mm needle was inserted caudal to cranial into the sacral canal under direct real-time longitudinal ultrasound guidance (Figure 1).

After confirming the absence of blood or cerebrospinal fluid through negative aspiration, 0.25% bupivacaine (0.5 ml/kg) was injected over one minute, with the injection observed in the longitudinal ultrasound image.

Dorsal Penile Nerve Block

**Figure 2:**
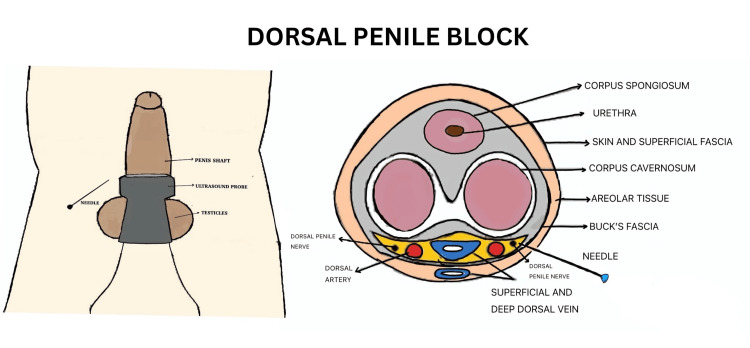
Dorsal penile nerve block

With the patient in a supine position, using aseptic precautions, a linear probe (6-13 MHz) was placed transversely at the root of the penis. The penis was held under gentle traction, and key anatomical structures, including the corpus cavernosum, corpus spongiosum, dorsal artery and vein, Buck’s fascia, and tunica albuginea, were identified in the transverse plane (Figure 2).

A 22G, 50 mm block needle (Stimuplex Ultra 360; B. Braun, Germany) was inserted from the lateral aspect of the penile root toward the dorsal section using the in-plane technique. The needle was advanced through the hyperechoic superficial fascia (dartos fascia) and Buck’s fascia and positioned between Buck’s fascia and the tunica albuginea, lateral to the dorsal artery. After confirming the absence of blood on negative aspiration, half of the calculated 0.2 ml/kg dose of 0.25% bupivacaine was injected under US guidance, ensuring proper distribution. The procedure was repeated on the opposite side of the penis.

Intraoperative analgesic requirements were managed with a bolus of 0.5 µg/kg fentanyl if more than 20% increase in heart rate (HR) or blood pressure was observed. The skin incision was made 15 minutes after the block. Hemodynamic monitoring was performed continuously throughout the procedure, with any adverse events promptly recorded and treated. HR, SpO_2_, mean arterial pressure (MAP) and respiratory rate (RR) were recorded along with intraoperative doses of fentanyl administered. To reverse the residual neuromuscular blockade, neostigmine (0.05 mg/kg) and glycopyrrolate (0.01 mg/kg) were administered intravenously at the end of the surgery. The supraglottic airway device was then removed, and once the patient regained consciousness, they were transferred to the post-anesthesia care unit (PACU). The FLACC pain scale was used for pain assessment at 0, 1, 2, 4, 6, 8, 12, and 24 hours postoperatively and an intravenous paracetamol dose of 15 mg/kg was given as rescue analgesia if the FLACC score was ≥4.

Statistical analysis

Data was entered into Microsoft Excel, version 2019 (Microsoft Corporation, Redmond, WA) and analyzed using IBM SPSS Statistics for Windows, version 21.0 (IBM Corp., Armonk, NY). Data was represented as frequency/percentage and summarized as mean ± SD/median. Categorical data was assessed using the chi-square test or Fisher's exact test; continuous data was compared using the Student's t-test or Mann-Whitney test. The results were considered statistically significant at p<0.05.

## Results

**Figure 3:**
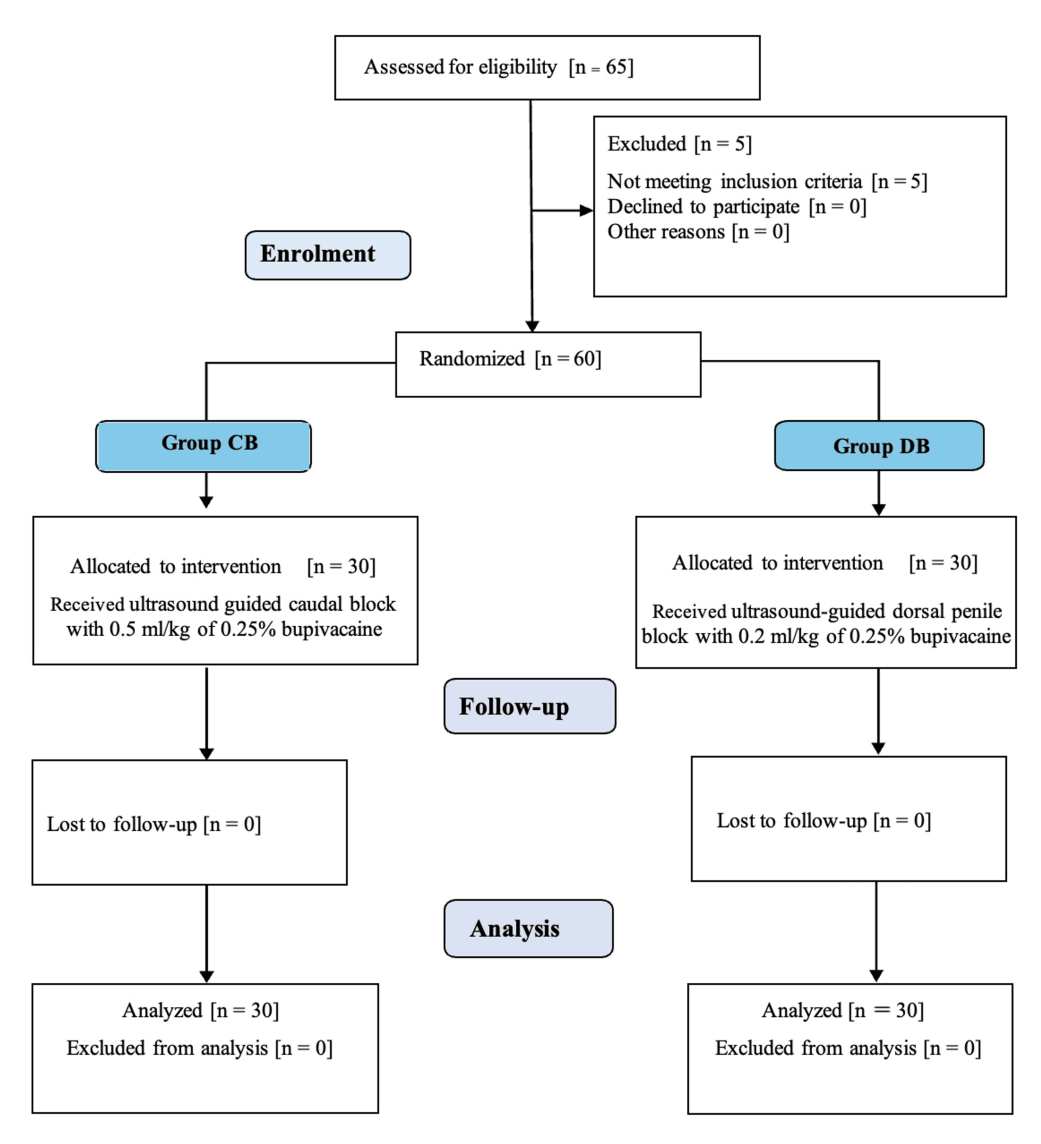
CONSORT diagram for patient selection and group allocation

A total of 60 patients were recruited, and all of them successfully completed the study (Figure 3).

Both groups were comparable in terms of demographic characteristics and surgical duration (Table 1).

**Table 1:** Demographic profile and duration of surgery n: number of patients; ASA PS: American Society of Anesthesiologists physical status; CB: caudal epidural block; DB: dorsal penile nerve block Data is expressed as mean ± SD and n (%), significant at p<0.05. ^*^Student's t-test, ^#^chi-square test

Parameters	Group		p-value
	CB (n = 30)	DB (n = 30)	
Age (years)	3.19 ± 1.22	3.06 ± 1.39	0.671^*^
Weight (kg)	14.38 ± 3.60	13.56 ± 3.78	0.393^*^
ASA PS			0.473^#^
I	24 (80%)	27 (90%)	
II	6 (20%)	3 (10%)	
Duration of surgery (minutes)	107.90 ± 37.23	96.94 ± 32.27	0.227^*^

The time to first rescue analgesia was 11.48 ± 6.13 hours in Group CB and 20.90 ± 6.49 hours in Group DB, with a statistically significant difference (p<0.001). There was a statistically significant difference in rescue analgesia requirements within 24 hours postoperatively, with 96.7% of patients in Group CB needing it compared to 23.3% in Group DB (p<0.001). In Group CB, 30%, 46.7%, and 10% of patients required one, two, and three doses of rescue analgesia, respectively, compared to 10%, 10%, and 3.3% in Group DB (p<0.001). Intraoperative fentanyl use was comparable between groups, required in 30% patients of Group CB (9.44 ± 3 µg) and 16.7% in Group DB (8 ± 4.47 µg) (Table 2).

**Table 2:** Comparison of rescue analgesia and intra-operative fentanyl uses n: number of patients; CB: caudal epidural block; DB: dorsal penile nerve block Data is expressed as mean ± SD and n (%), significant at p<0.05. ^*^Student’s t-test, ^#^chi-square test, ^$^Fisher’s exact test

Variable	Group CB (n = 30)	Group DB (n = 30)	p-value
Time to first rescue analgesia (in hours)	11.48 ± 6.13	20.90 ± 6.49	<0.001^*^
Rescue analgesia required	29 (96.7%)	7 (23.3%)	<0.001^#^
Doses of rescue analgesia			<0.001^$^
None	1 (3.3%)	23 (76.7%)	
1 dose	12 (30%)	3 (10%)	
2 doses	14 (46.7%)	3 (10%)	
3 doses	3 (10%)	1 (3.3%)	
Number of doses of rescue analgesia	1.61 ± 0.72	0.39 ± 0.80	<0.001^*^
Intra-operative fentanyl dose	9.44 ± 3.00	8.00 ± 4.47	0.421^*^

The FLACC pain scale scores were comparable at baseline and up to two hours. From four hours onward, Group CB showed consistently higher scores compared to Group DB: 0.26 ± 0.51 vs. 0 (p=0.006) at 4 hours, 0.74 ± 0.73 vs. 0.23 ± 0.50 (p=0.001) at 6 hours, 1.42 ± 1.06 vs. 0.55 ± 0.62 (p<0.001) at 8 hours, 1.74 ± 1.32 vs. 0.65 ± 0.91 (p<0.001) at 12 hours, and 1.58 ± 0.56 vs. 0.58 ± 0.72 (p<0.001) at 24 hours (Table 3). The mean FLACC score was 0.72 ± 0.29 in Group CB and 0.25 ± 0.22 in Group DB, with the difference being statistically significant (p<0.001).

**Table 3:** Comparison of FLACC scores FLACC: Face, Legs, Activity, Cry and Consolability scale; CB: caudal epidural block; DB: dorsal penile nerve block Data is expressed as mean ± SD, significant at p<0.05. ^*^Student’s t-test

Variables	Categories	Group CB (n = 30)	Group DB (n = 30)	p-value
FLACC	0 hour postoperative	0.00 ± 0.00	0.00 ± 0.00	-
	1 hour postoperative	0.00 ± 0.00	0.00 ± 0.00	-
	2 hours postoperative	0.00 ± 0.00	0.00 ± 0.00	-
	4 hours postoperative	0.26 ± 0.51	0.00 ± 0.00	0.006^*^
	6 hours postoperative	0.74 ± 0.73	0.23 ± 0.50	0.001^*^
	8 hours postoperative	1.42 ± 1.06	0.55 ± 0.62	<0.001^*^
	12 hours postoperative	1.74 ± 1.32	0.65 ± 0.91	<0.001^*^
	24 hours postoperative	1.58 ± 0.56	0.58 ± 0.72	<0.001^*^
	Average FLACC score	0.72 ± 0.29	0.25 ± 0.22	<0.001^*^

## Discussion

This study aimed to compare the effectiveness of postoperative analgesia between caudal epidural block and dorsal penile nerve block in hypospadias surgery. The time to first rescue analgesia was significantly longer in Group DB compared to Group CB. The requirement for rescue analgesia within 24 hours was significantly lower in Group DB than in Group CB. FLACC pain scores were significantly lower in Group DB from four hours onwards postoperatively, suggesting better sustained pain control.

The ideal analgesic regimen employs a multimodal approach to minimize postoperative stress, enhance recovery, and provide safe and effective pain relief. CB and DB are popular regional anesthesia techniques for hypospadias surgery, with CB being the most used approach for postoperative pain relief in pediatric lower abdomen and urogenital surgeries [12].

Limited studies have directly compared CB and DB in patients undergoing hypospadias surgery [2,13]. Our study findings align with those reported by Ozen and Yigit who compared US-guided dorsal penile nerve block versus caudal epidural block in pediatric patients undergoing hypospadias surgery [2]. They found that DB provided significantly prolonged analgesia (mean duration: 20 hours vs. 12 hours in the CB group), lower pain scores at the 12th postoperative hour (p=0.003), and a reduced need for additional postoperative analgesia (p<0.001). Furthermore, parental satisfaction regarding the child's postoperative condition was higher in the DB group (100%) compared to the CB group (69.2%).

Beyaz compared caudal block and dorsal penile nerve block using levobupivacaine for circumcision surgery in children and found no statistically significant difference in the time to first analgesic requirement between the two groups [14]. This outcome may be due to the penile block being performed using a landmark-based technique instead of ultrasound guidance.

Kundra et al. reported that DB offered prolonged postoperative analgesia and reduced opioid use compared to CB [15]. Similarly, our study found that DB provided longer lasting pain relief with a lower need for additional analgesia compared to CB. However, while Kundra et al. reported a mean postoperative analgesia duration of 5 hours with DB, we observed a significantly longer duration of 21 hours in our study. This difference may be attributed to the precise administration technique used in our study, where DB was performed under ultrasound guidance. Particular attention was given to ensuring that Buck’s fascia was penetrated and the local anesthetic was accurately deposited near the dorsal penile nerve. This meticulous approach, in line with the existing literature [16], likely contributed to the prolonged analgesic effect observed in our study.

The FLACC pain scale indicated significantly higher scores in the CB group at 4, 6, 8, 12, and 24 hours. The average FLACC score was also markedly higher in the CB group. Similar findings were reported by Ozen and Yigit, who observed higher mean and 12th-hour pain scores in the CB group compared to the DB group [2].

Ahmed et al. reported significantly higher pain levels in the caudal block group compared to the pudendal nerve block group at the 6-hour and 12-hour time points (p=0.017). However, no significant difference was noted at 18 hours [3].

Yiğit et al. reported a lower incidence of complications in the DB group when compared to the caudal block group, which included meatal stenosis and fistula formation with this difference being statistically significant (p=0.03) [11].

Bansal et al. compared US‑guided sacral erector spinae plane block (ESPB) and caudal epidural block among patients undergoing hypospadias repair and found US-guided sacral ESPB to be a superior analgesia method with a higher mean time to rescue analgesia, lower mean FLACC score and lower mean postoperative analgesia consumption [17].

Aksu et al. compared ultrasound-guided dorsal penile nerve block and neurostimulator-guided pudendal nerve block (NSG PNB) for postoperative analgesia in pediatric hypospadias surgery. They found that DB provided significantly longer pain relief, with the time to first analgesic requirement being 32.29 ± 5.47 hours in the DB group compared to 21.13 ± 3.53 hours in the PNB group (p<0.001). FLACC scores were significantly lower in the DB group at 18 and 24 hours and at the time of first analgesic requirement. Total doses of both paracetamol and tramadol were significantly lower in the DB group, and parental satisfaction scores for analgesia were higher. They concluded that DB is a more effective and longer lasting analgesic technique than NSG PNB in children undergoing hypospadias surgery, with reduced analgesic requirements [18].

This study also has some limitations. This was a single-center study with a small sample size, limiting its generalizability. Findings are specific to children aged one to six years undergoing hypospadias surgery and may not apply to other age groups. Additionally, the lack of follow-up beyond 24 hours prevented assessment of long-term analgesic effects.

## Conclusions

We conclude that ultrasound-guided DB was more effective than CB for postoperative analgesia in pediatric hypospadias surgery. There was a reduced need for rescue analgesia in the DB group. Moreover, the mean FLACC score was lower in the DB group. However, further studies with larger sample sizes and longer follow-ups are needed to corroborate our study findings.
